# ACTION (acute coronary treatment and intervention outcomes network) registry-GWTG (get with the guidelines) risk score predicts long-term mortality in acute myocardial infarction

**DOI:** 10.18632/oncotarget.21741

**Published:** 2017-10-11

**Authors:** Tongtong Yu, Chunyang Tian, Jia Song, Dongxu He, Zhijun Sun, Zhaoqing Sun

**Affiliations:** ^1^ Department of Cardiology, Shengjing Hospital of China Medical University, Shenyang, Liaoning, P.R.China

**Keywords:** ACTION, risk score, long-term mortality, acute myocardial infarction, percutaneous coronary intervention

## Abstract

This study aimed to test the predictive performance of the updated ACTION, GRACE, and CADILLAC risk scores (RS’s) for long-term mortality in patients with acute myocardial infarction (AMI) undergoing percutaneous coronary intervention (PCI). The study included individuals from 2 independent cohorts: derivation cohort (*N* = 1901) and validation cohort (*N* = 728). From the derivation cohort, we used Cox regression analysis to determine that the updated ACTION, GRACE, and CADILLAC RS's were associated with long-term mortality. The concordance (C) statistics of the 3 RS's were 0.682, 0.703 and 0.734, respectively. We used the validation cohort to validate the results. Moreover, the discriminatory performance of the updated ACTION RS for predicting long-term mortality in both the respective derivation and validation cohorts was similar to the discriminatory performance of the GRACE and CADILLAC RS's (ACTION vs. GRACE: *z* = 0.684, *p* = 0.494; ACTION vs. CADILLAC: *z* = 1.638, *p* = 0.101) and (ACTION vs. GRACE: *z* = 0.460, *p* = 0.646; ACTION vs. CADILLAC: *z* = 0.290, *p* = 0.772). Despite their development over a decade ago, GRACE and CADILLAC RS's maintain good performance for predicting the long-term mortality of AMI patients undergoing PCI. As a new risk model, the updated ACTION RS also predicts long-term mortality, and its discriminatory performance is similar to that of the GRACE and CADILLAC RS’s.

## INTRODUCTION

Although advances have been made in the treatment strategies and management of patients with acute myocardial infarction (AMI), some patients still have poor outcomes [1–4]. Early risk stratification is important for such patients. Patients found to be at high risk by early risk stratification should receive appropriate clinical treatment and long-term follow up. The Acute Coronary Treatment and Intervention Outcomes Network (ACTION) Registry-Get With The Guidelines (GWTG) AMI mortality model and risk score (ACTION) were introduced in 2011 to predict in-hospital mortality [5]. The simple risk model was subsequently externally validated [6]. The existing ACTION risk model was updated in 2016 [7]. The C statistic of the updated ACTION risk model was 0.88, with good calibration. However, no published study has focused on the value of the risk model for predicting long-term mortality. An extensive assessment of the prognostic value of the risk model for patients with AMI undergoing percutaneous coronary intervention (PCI) that validates the risk model has not been performed.

However, over the last few decades, many other risk models that facilitate assessment of the long-term outcomes of AMI patients with or without PCI have been introduced. Among these risk models, the Global Registry of Acute Coronary Events (GRACE) clinical risk scoring system is most frequently used and recommended [1–4]. It was derived from a very large ‘real-world’, worldwide study of patients with the entire spectrum of acute coronary syndromes (ACS’s), and can predict in-hospital [8], 6-month [9], and even longer-term (up to 4 years) [10] mortality after ACS to help us identify high-risk patients. The CADILLAC risk score was derived from participants of the Controlled Abciximab and Device Investigation to Lower Late Angioplasty Complications (CADILLAC) trial [11]. It was found to provide accurate predictions of in-hospital and 1-year mortality of AMI patients undergoing primary PCI. Both risk models have been in use for more than a decade; however, the treatment and management of patients with AMI has greatly improved over this past decade. Therefore, these older risk models should be validated in recent patient cohorts. In addition, to the best of our knowledge, no study has been published that compares the prognostic accuracy of GRACE and CADILLAC with new risk models, such as the updated ACTION risk model, for the long-term outcome of patients with AMI who PCI.

In this retrospective cohort study, we aimed to determine if the updated ACTION risk score is a useful method for predicting long-term mortality in AMI patients undergoing PCI. We then used an independent prospective cohort to validate our findings. We also used both cohorts to test the predictive performance of the GRACE and CADILLAC risk scores and to compare the prognostic accuracy of the updated ACTION risk score with the prognostic accuracy of the GRACE and CADILLAC risk scores.

## RESULTS

### Derivation and validation cohorts

**Figure 1:**
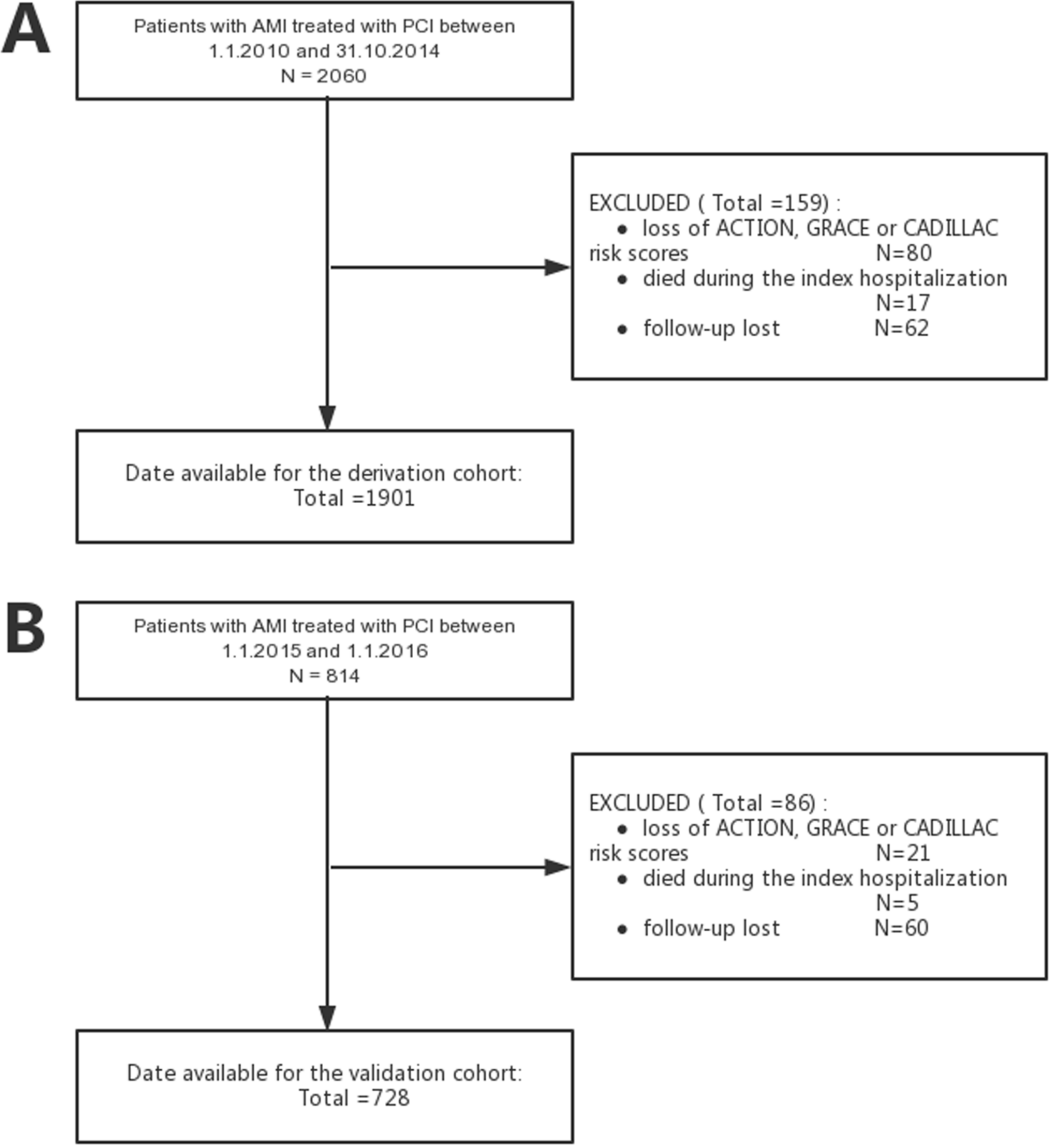
The population flow diagram for the derivation cohort (A) and the validation cohort (B)

As shown by the population flow diagram (Figure 1), the final numbers of patients in the derivation and validation cohorts were 1901 and 728 patients, respectively. Table 1 shows the baseline characteristics of the 2 cohorts. Participants in the validation cohort had higher left ventricular ejection fractions (LVEFs) and greater creatinine clearance and lower ACTION, GRACE, or CADILLAC risk scores than participants in the derivation cohort. The validation cohort had a lower prevalence of anemia, three-vessel disease, use of intra-aortic balloon pump, use of angiotensin-converting enzyme inhibitors/angiotensin receptor blockers, and use of beta-blockers than the derivation cohort. The validation cohort showed higher prevalence of prior MI, prior PCI, use of glycoprotein IIb/IIIa inhibitors, and statins than the derivation cohort. However, other characteristics such as age and gender distributions were comparable between cohorts (Table 1).

**Table 1:** Baseline characteristics for the derivation and validation cohorts, median (IQR), or N (%), or means ± SD

Variable	Derivation Cohort, *n* = 1901	Validation Cohort, *n* = 728	*p*-Value
Demographics			
Age, yrs	61.9 ± 12.0	61.1 ± 11.7	0.115
Female	555 (29.2)	205 (28.2)	0.600
Medical history			
History of Diabetes Mellitus	681 (35.8)	237 (32.6)	0.116
History of Hypertension	1034 (54.4)	406 (55.8)	0.526
History of renal dysfunction	180 (9.5)	70 (9.6)	0.909
After cardiac arrest	76 (4.0)	30 (4.1)	0.458
In cardiogenic shock	28 (1.5)	14 (1.9)	0.410
In HF	101 (5.3)	52 (7.1)	0.073
History of MI	108 (5.7)	59 (8.1)	0.023
Prior PCI	114 (6.0)	61 (8.4)	0.028
Prior peripheral arterial disease	17 (0.9)	7 (1.0)	0.871
Anemia	556 (29.2)	181 (24.9)	0.025
Presentation			
SBP on admission, mm Hg	133.0 ± 22.8	132.8 ± 22.4	0.858
Heart rate on admission, beats/min	77.2 ± 14.9	77.0 ± 14.5	0.787
LVEF, %	56.0 ± 9.8	57.3 ± 8.9	0.003
ACTION risk score	35.7 ± 11.2	34.4 ± 10.7	0.005
GRACE risk score	136.7 ± 34.4	132.3 ± 32.5	0.003
CADILLAC risk score	2 (0, 4)	2 (0, 4)	0.001
Diagnosis on admission			0.096
NSTEMI	886 (46.6)	313 (43.0)	
STEMI	1015 (53.4)	415 (57.0)	
Troponin-I on admission, ng/mL	6.40 (0.66, 32.41)	6.85 (0.67, 44.09)	0.175
Creatinine clearancec	89.3 ± 39.4	93.0 ± 38.0	0.029
PCI details			
Left main disease	173 (9.1)	62 (8.5)	0.639
Three-vessel disease	604 (31.8)	198 (27.2)	0.023
Intra-aortic Balloon Pump	116 (6.1)	26 (3.6)	0.010
TIMI flow grade 3 post PCI	1892 (99.5)	723 (99.3)	0.501
Use of glycoprotein IIb/IIIa inhibitor	669 (35.2)	334 (45.9)	< 0.001
Medical treatment at discharge			
Aspirin	1882 (99.0)	726 (99.7)	0.062
Clopidogrel	1842 (96.9)	715 (98.2)	0.064
Ticagrelor	25 (1.3)	7 (1.0)	0.459
Statin	1803 (94.8)	722 (99.2)	< 0.001
Angiotensin-converting enzyme inhibitors / Angiotensin receptor blockers	1091 (57.4)	313 (43.0)	< 0.001
Beta-blockers	1021 (53.7)	297 (40.8)	< 0.001

HF, heart failure; MI, myocardial infarction; LVEF, left ventricular ejection fraction; PCI, percutaneous coronary intervention; STEMI, ST-segment elevation myocardial infarction; NSTEMI, non-ST-segment elevation myocardial infarction.

### Development of updated ACTION risk score as a predictive parameter of outcome

Over a mean follow up of 32 months, there were 64 events (3.4% of 1901 cases) of all-cause mortality in the derivation cohort. Cox regression analysis of the updated ACTION, GRACE, and CADILLAC risk scores, which were used as continuous variables, were significantly predictive of long-term mortality (Table 2). When categorized into groups, all 3 risk scores remained significantly predictive of long-term mortality (Table 2). Compared with the very-low-risk ACTION score, the patient groups with low-risk, intermediate-risk, high-risk, and very-high-risk ACTION scores had a substantially higher risk of death (Table 2). Compared with the low-risk GRACE and CADILLAC scores, the respective patient groups with GRACE and CADILLAC intermediate-risk and high-risk scores had a substantially higher risk of death.

**Table 2:** Effects of ACTION, GRACE and CADILLAC risk scores on the outcome in COX analysis for the derivation and validation cohorts

	HR	95% confidence interval	*p*-Value
Derivation Cohort			
ACTION risk score (continue)	1.061	1.041–1.082	< 0.001
GRACE risk score (continue)	1.022	1.015–1.028	< 0.001
CADILLAC risk score (continue)	1.334	1.243–1.432	< 0.001
ACTION risk score (categories)			
Very Low (0–29)	1.00		< 0.001
Low (30–39)	3.005	1.109–8.148	0.031
Intermediate (40–49)	5.274	1.969–14.130	0.001
High (50–59)	8.721	3.072–24.758	< 0.001
Very High (> 59)	24.482	8.503–70.493	< 0.001
GRACE risk score (categories)			
Low (0–88)	1.00		< 0.001
Intermediate (89–118)	2.886	1.205–6.910	0.017
High (> 118)	6.344	2.837–14.186	< 0.001
CADILLAC risk score (categories)			
Low (0–2)	1.00		< 0.001
Intermediate (3–5)	2.056	1.039–4.070	0.039
High (> 5)	7.712	4.268–13.935	< 0.001
Validation Cohort			
ACTION risk score (continue)	1.063	1.032–1.095	< 0.001
GRACE risk score (continue)	1.024	1.014–1.035	< 0.001
CADILLAC risk score (continue)	1.394	1.207–1.611	< 0.001
ACTION risk score (categories)			
Very Low (0–29)	1.00		< 0.001
Low (30–39)	4.868	1.052–22.530	0.043
Intermediate (40–49)	9.061	1.985–41.354	0.004
High (50–59)	12.916	2.607–63.997	0.002
Very High (> 59)	32.339	4.555–229.601	0.001
GRACE risk score (categories)			
Low (0–88)	1.00		< 0.001
Intermediate (89–118)	8.779	1.112–69.296	0.039
High (> 118)	20.173	2.701–150.692	0.003
CADILLAC risk score (categories)			
Low (0–2)	1.00		< 0.001
Intermediate (3–5)	4.149	1.720–10.011	0.002
High (> 5)	6.881	2.582–18.336	< 0.001

**Figure 2:**
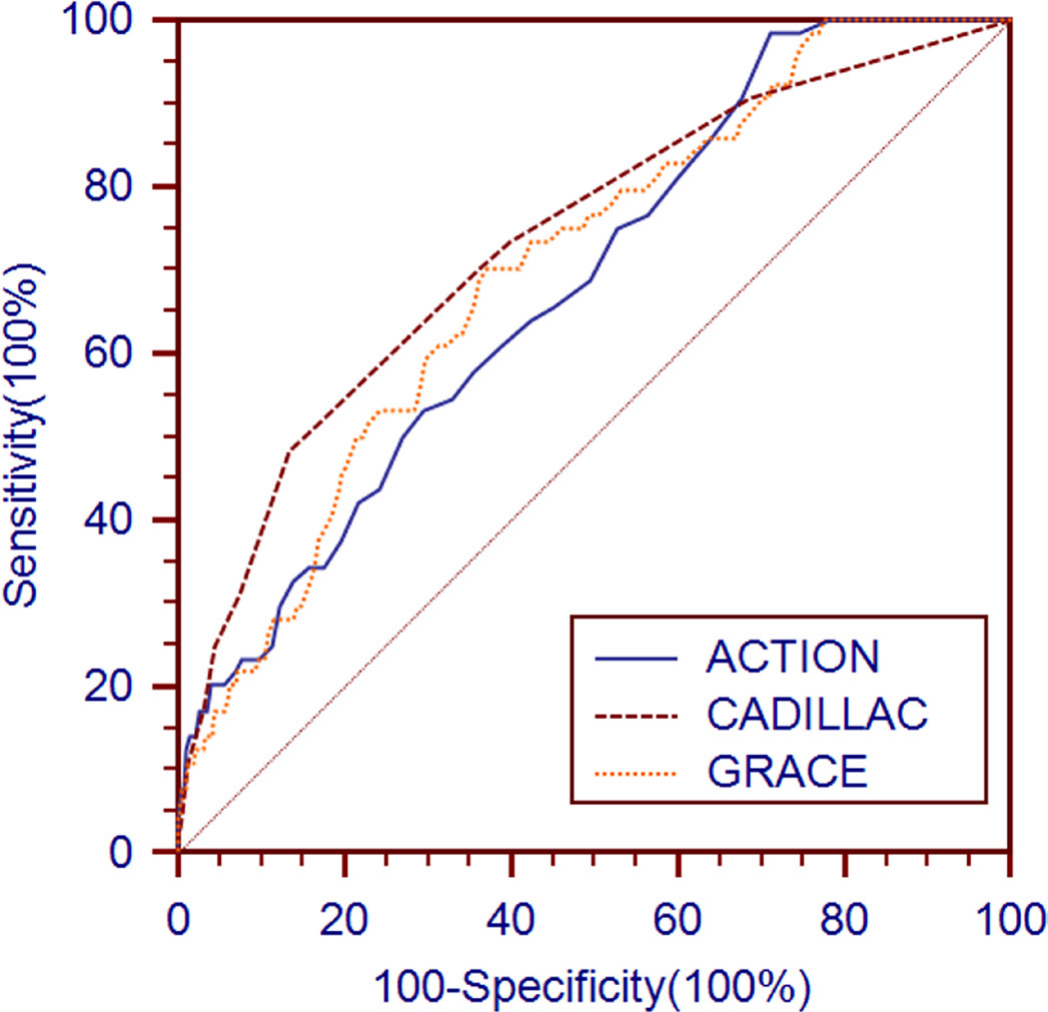
Receiver operating characteristic curves of the updated ACTION, GRACE and CADILLAC risk scores for long-term mortality prediction in the derivation cohort

The Hosmer-Lemeshow (HL) goodness-of-fit test indicated that the 3 risk models all showed good calibration for predicting death in the derivation cohort (Table 3). The C-statistics of the updated ACTION, GRACE, and CADILLAC risk scores for long-term mortality were 0.682, 0.703 and 0.734, respectively (Table 3 and Figure 2).

**Table 3:** Three scoring systems performance for the prognosis prediction in the derivation and validation cohorts

	Discrimination				Calibration
	C-Statistic	Standard error	*p*-Value	95% CI	H-L *p*-Value
Derivation Cohort					
ACTION risk score	0.682	0.0311	< 0.001	0.660–0.703	0.109
GRACE risk score	0.703	0.0306	< 0.001	0.681–0.723	0.135
CADILLAC risk score	0.734	0.0332	< 0.001	0.714–0.754	0.946
Validation Cohort					
ACTION risk score	0.713	0.0446	< 0.001	0.679–0.746	0.121
GRACE risk score	0.741	0.0424	< 0.001	0.707–0.772	0.297
CADILLAC risk score	0.731	0.0427	< 0.001	0.697–0.762	0.107

### Validation of updated ACTION risk score as a predictive parameter for outcome

The all-cause mortality of the validation cohort over a 1-year follow up was 4.0% (29 of 728 cases). Cox regression analysis of the updated ACTION, GRACE, and CADILLAC risk scores, which were used as continuous variables were significantly predictive of long-term mortality (Table 2). When categorized into groups, all 3 risk scores remained significantly predictive of long-term mortality (Table 2). Compared with the very-low-risk ACTION score, the patient groups with low-risk, intermediate-risk, high-risk, and very-high-risk ACTION scores had a substantially higher risk of death (Table 2). Compared with the low-risk GRACE and CADILLAC scores, the respective patient groups with GRACE and CADILLAC intermediate-risk and high-risk scores had a substantially higher risk of death.

**Figure 3:**
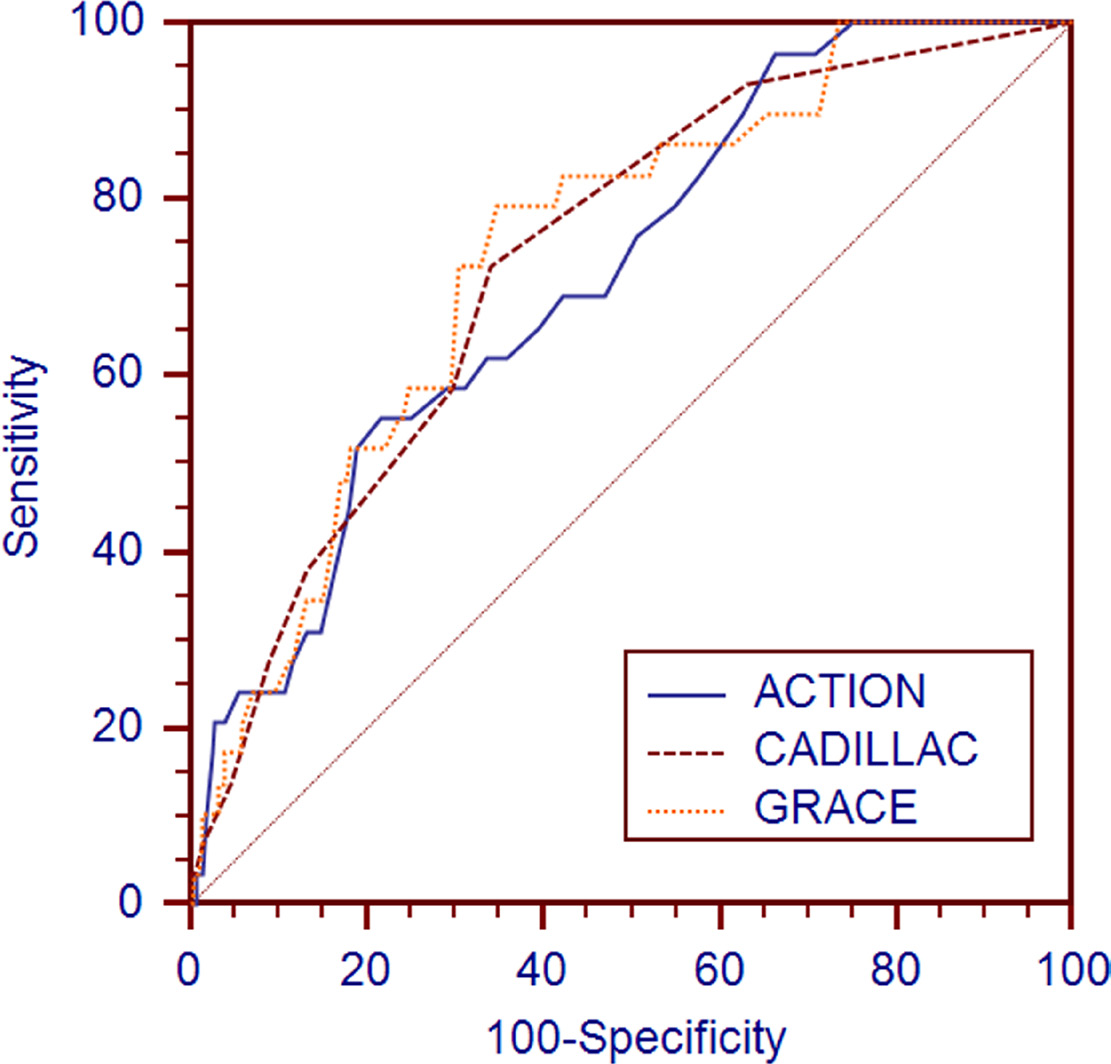
Receiver operating characteristic curves of the updated ACTION, GRACE and CADILLAC risk scores for long-term mortality prediction in the validation cohort

The HL goodness-of-fit test indicated that the 3 risk models all showed good calibration for predicting death in the validation cohort (Table 3). The C-statistics of the updated ACTION, GRACE and CADILLAC risk scores for long-term mortality were 0.713, 0.741 and 0.731, respectively (Table 3 and Figure 3).

### Prognostic accuracy of updated ACTION, GRACE, and CADILLAC risk scores

**Figure 4:**
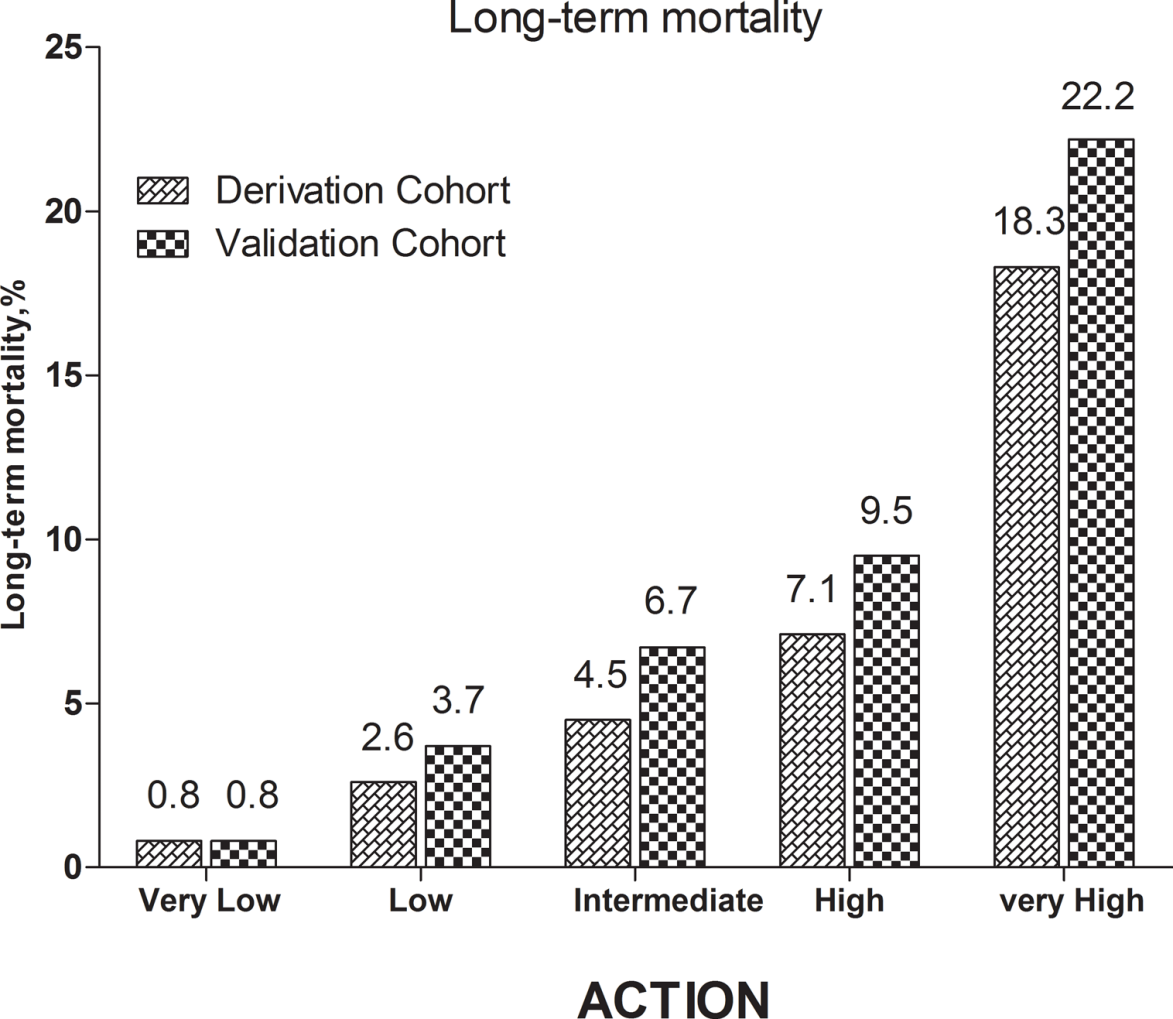
Long-term mortality in different risk stratifications according to the updated ACTION risk score in the both cohorts

**Figure 5:**
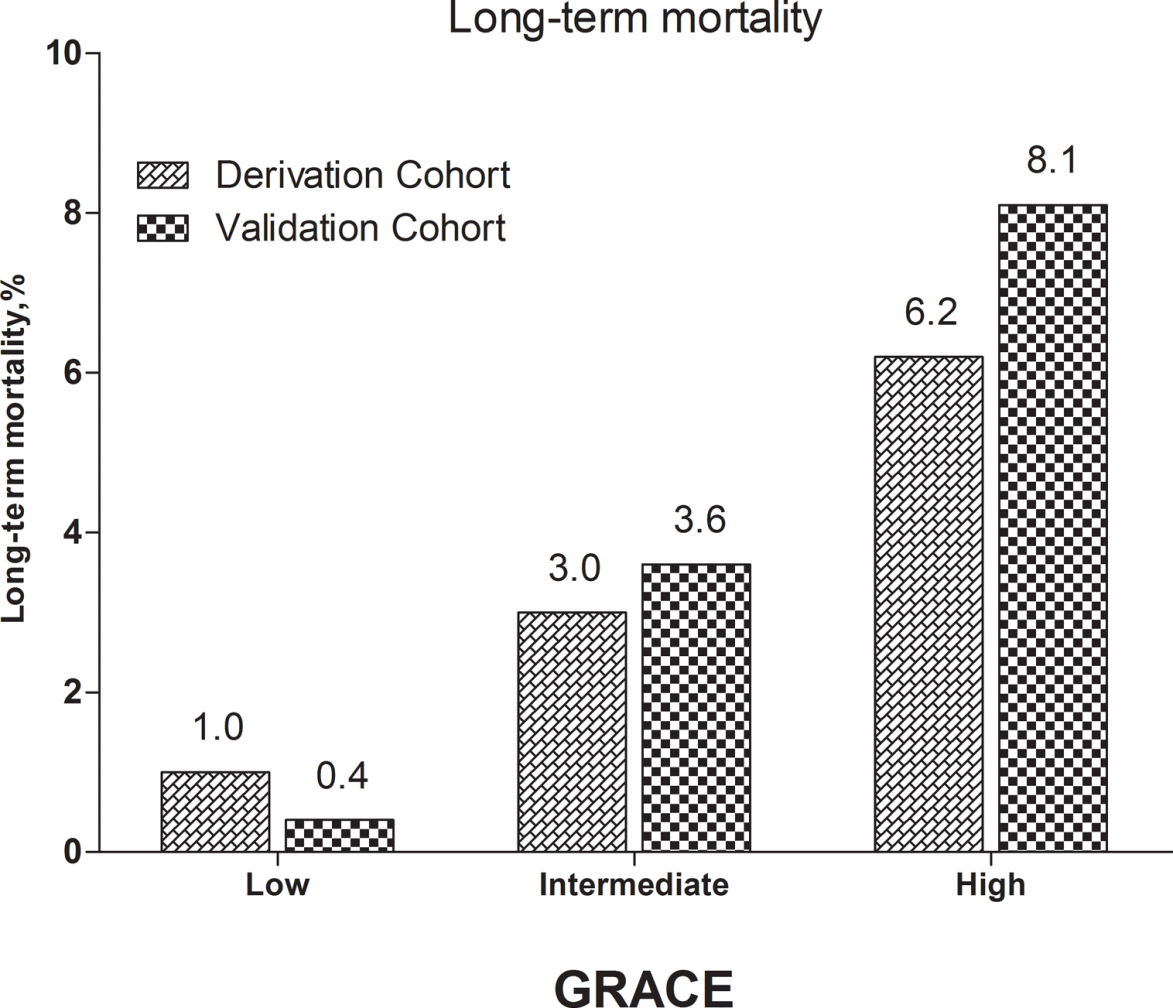
Long-term mortality in different risk stratifications according to the GRACE risk score in the both cohorts

**Figure 6:**
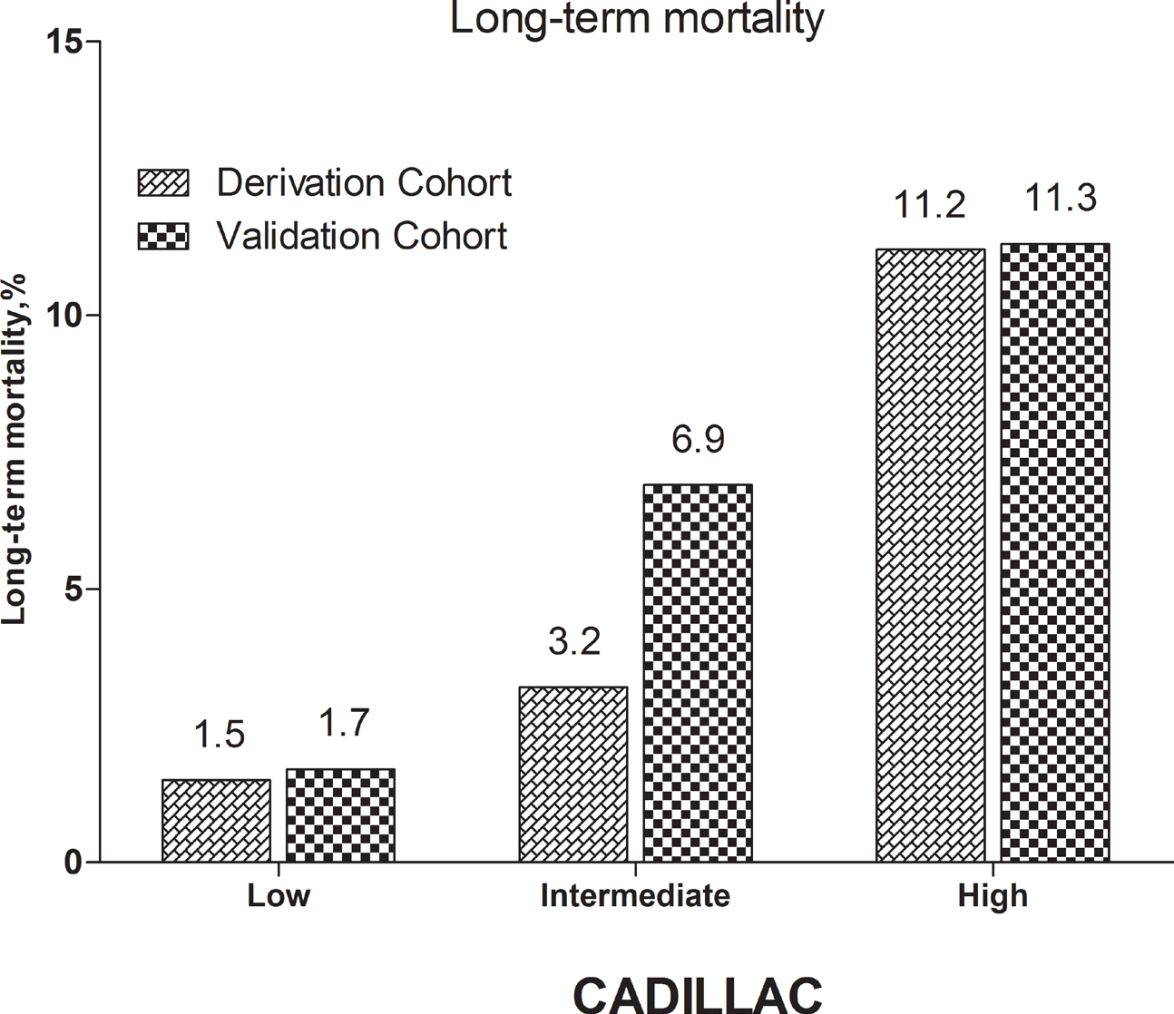
Long-term mortality in different risk stratifications according to the CADILLAC risk score in the both cohorts

The stratified risk scores of the 3 systems could all identify the high-risk patients in both cohorts as follows: updated ACTION risk score, Figure 4 shows 5 subgroups in the derivation cohort (*p* < 0.001) and 5 subgroups in the validation cohort, (*p* < 0.001); GRACE risk score, Figure 5 shows 3 subgroups in the derivation cohort (*p* < 0.001) and 3 subgroups in the validation cohort (*p* < 0.001); CADILLAC risk score, Figure 6 shows 3 subgroups in the derivation cohort (*p* < 0.001) and 3 subgroups in the validation cohort (*p* < 0.001).

For the derivation cohort, the discriminatory performance of the updated ACTION risk score was similar to the discriminatory performances of the GRACE and CADILLAC risk scores for predicting long-term all-cause mortality (ACTION *vs.* GRACE: *z* = 0.684, *p* = 0.494; ACTION *vs.* CADILLAC: *z* = 1.638, *p* = 0.101) (Table 4). For the validation cohort, the discriminatory performance of the updated ACTION risk score was similar to the discriminatory performances of the GRACE and CADILLAC risk scores for predicting long-term all-cause mortality (ACTION *vs.* GRACE: *z* = 0.460, *p* = 0.646; ACTION *vs.* CADILLAC: *z* = 0.290, *p* = 0.772) (Table 4).

**Table 4:** Comparisons of the predictive accuracy of three scoring systems for the prognosis prediction in the derivation and validation cohorts

	Difference	Z	*p*-Value
Derivation Cohort			
ACTION vs. GRACE	0.0206	0.684	0.494
ACTION vs. CADILLAC	0.0525	1.638	0.101
CADILLAC vs. GRACE	0.0319	0.788	0.431
Validation Cohort			
ACTION vs. GRACE	0.0273	0.460	0.646
ACTION vs. CADILLAC	0.0171	0.290	0.772
CADILLAC vs. GRACE	0.0102	0.224	0.823

For both cohorts, the discriminatory performance of the GRACE risk score was similar to the discriminatory performance of the CADILLAC risk score for predicting long-term all-cause mortality (derivation cohort, GRACE *vs.* CADILLAC: *z* = 0.788, *p* = 0.431; validation cohort, GRACE *vs.* CADILLAC: *z* = 0.224, *p* = 0.823) (Table 4).

### Predicted and observed mortality rates of the different patient subgroups in the validation cohort

The predicted and observed mortality rates of the different patient subgroups in the validation cohort were compared (Table 5). These subgroups included the following: age < 65 yrs, age ≥ 65 yrs, male, female, diabetes mellitus, no diabetes mellitus, hypertension, no hypertension, ST-segment elevation myocardial infarction (STEMI), non-ST-segment elevation myocardial infarction (NSTEMI), three-vessel disease, and no three-vessel disease. There were no significant differences between the predicted and observed mortality rates of all the subgroups for the updated ACTION, GRACE and CADILLAC risk models (Table 5).

**Table 5:** Comparisons of the predicted and observed mortality rate in different patient subgroups for the validation cohort

	Observed mortality rate (%)	ACTION risk model		GRACE risk model		CADILLAC risk model	
		Predicted mortality rate (%)	*p*	Predicted mortality rate (%)	*p*	Predicted mortality rate (%)	*p*
Age ≥ 65	5.9	5.3	0.756	6.1	0.851	5.9	1.000
Age < 65	3.1	3.3	0.799	2.8	0.692	2.9	0.793
Male	3.6	3.8	0.813	3.7	0.905	3.6	1.000
Female	4.9	4.5	0.673	4.6	0.752	5.0	0.918
DM	3.0	3.7	0.384	3.8	0.324	4.6	0.061
No DM	4.5	4.1	0.659	4.1	0.659	3.7	0.367
Hypertension	5.2	4.0	0.200	4.1	0.243	4.2	0.291
No Hypertension	2.5	4.0	0.059	3.8	0.096	3.7	0.122
NSTEMI	4.2	3.0	0.150	5.0	0.393	4.4	0.826
STEMI	3.9	4.8	0.324	3.2	0.398	3.7	0.815
TVD	4.5	4.3	0.827	4.6	0.915	6.0	0.133
No TVD	3.8	3.9	0.907	3.8	1.000	3.2	0.465

DM, Diabetes Mellitus; TVD, Three-vessel disease; STEMI, ST-segment elevation myocardial infarction; NSTEMI, non-ST-segment elevation myocardial infarction

## DISCUSSION

To the best of our knowledge, this is the first study to investigate the predictive value of the updated ACTION risk model for long-term mortality in AMI patients undergoing PCI. The main findings were as follows: (1) the updated ACTION risk model predicted the long-term mortality of patients with AMI undergoing PCI; (2) despite their development over a decade ago, the GRACE and CADILLAC risk scores still maintain good performance for predicting the long-term risk of mortality of AMI patients undergoing PCI in 2 recent cohorts; (3) the discriminatory performance of the updated ACTION risk model was similar to the discriminatory performance of the GRACE and CADILLAC scoring systems for predicting long-term mortality.

The ACTION risk model was developed and validated to predict the risk of in-hospital mortality in 2011 in order to improve performance quality and guide investigators studying patients with STEMI or NSTEMI who were treated in routine clinical practice [5]. The ACTION risk model was developed from data in the ACTION Registry-GWTG database, which included a very large number of consecutive AMI patients from various institutions and regions across the United States. The developers found that the following factors showed the highest independent significance for in-hospital mortality: age, baseline serum creatinine, systolic blood pressure, elevated levels of troponin, heart failure and/or cardiogenic shock at presentation, ST-segment changes, heart rate, and prior peripheral artery disease. The ACTION risk model showed very good discrimination, with C statistics of 0.85 and 0.84 in the derivation and validation cohorts, respectively, and very good overall calibration and very good calibration in key subgroups. In 2012, Raposeiras-Roubín et al compared the in-hospital prognostic values of the original and updated GRACE and the ACTION risk scores in 4497 consecutive ACS patients admitted to a single center in Spain [6]. The risk scores all showed good discrimination for in-hospital death (C-statistics: 0.91, 0.90 and 0.90, respectively) with optimal calibration (HL p: 0.42, 0.50, and 0.47, respectively). In 2016, data from January 2012 through December 2013 in the ACTION Registry-GWTG database was used to update the ACTION risk model [7]. Several changes in risk adjustment were incorporated into the new model. Firstly, the updated model included presentation after cardiac arrest, which was not available at the time the old model was built. Secondly, the updated model included creatinine clearance instead of serum creatinine level. Lastly, heart failure and cardiogenic shock at the time of presentation were separated. The new variables were all independently associated with in-hospital mortality and showed increased predictive power. However, the updated ACTION risk model has not yet been validated by an external cohort. To the best of our knowledge, this is the first study that has demonstrated the long-term prognostic value of the updated ACTION risk model in patients with AMI undergoing PCI. With ACTION risk scores as continuous variables, we found that the risk of long-term mortality increased by 6.1% per increase in unit of the ACTION risk score in the derivation cohort (hazard ratio [HR] = 1.061, 95% confidence interval [CI] 1.041–1.082, *p* < 0.001), or 6.3% per increase in unit elevation of the ACTION risk score in the validation cohort (HR = 1.063, 95% CI 1.032–1.095, *p* < 0.001). As shown in Table 2, the ACTION risk score still maintained an independent and significant positive correlation with all-cause mortality as a categorical variable.

Based on the updated ACTION risk model, a simplified integer score performed well and could easily identify the high-risk patients; patients in the very-high-risk ACTION risk score group had the highest all-cause mortality in both cohorts (Figure 4). The model showed good discriminatory performance with good calibration in both the validation and derivation cohorts (C-statistics, 0.682, 0.713, respectively; H-L *p*-values = 0.109, 0.121, respectively) (Tables 3 and 5). The updated ACTION risk model might serve as the cornerstone for prospective risk stratification at the point of patient care. Moreover, the prognostic accuracy of the updated ACTION risk model was similar to the prognostic accuracy of the GRACE and CADILLAC scoring systems for predicting long-term mortality in patients with AMI undergoing PCI, although there were subtle differences in the C-statistics (derivation cohort: ACTION *vs.* GRACE: *z* = 0.684, *p* = 0.494; ACTION *vs.* CADILLAC: *z* = 1.638, *p* = 0.101; validation cohort: ACTION *vs.* GRACE: *z* = 0.460, *p* = 0.646; ACTION *vs.* CADILLAC: *z* = 0.290, *p* = 0.772).

The GRACE risk score was first developed to assessing in-hospital [8] and 6-month [9] mortality after ACS. It was soon verified by many external cohorts from various countries and regions [6, 18–22]. Furthermore, it was extended to predict angiographic severity and contrast-induced nephropathy in patients with ACS [23, 24]. It could also identify those high-risk patients who required urgent or elective PCI [25]. Our study again confirmed that the GRACE risk score could predict the long-term mortality of patients with AMI undergoing PCI (derivation cohort: HR = 1.022, 95% CI 1.015 –1.028, *p* < 0.001; validation cohort: HR = 1.024, 95% CI 1.014–1.035, *p* < 0.001). Also, as a categorical variable, the GRACE risk score was independently associated with all-cause mortality and could easily identify high-risk patients; patients in the high-risk GRACE risk score groups of both cohorts showed the highest all-cause mortality (Table 2 and Figure 5). In our study, the GRACE risk score still had good prognostic accuracy with good calibration in both the validation and derivation cohorts (C-statistics: 0.703, 0.741, respectively; H-L *p*-Value = 0.135, 0.297, respectively) (Tables 3 and 5). The good discriminatory performance of GRACE was expected. The GRACE risk scoring system is unparalleled, since it was derived from a large worldwide registry of ‘real-world’ patients with the entire spectrum of ACS. However, in our study, the predictive accuracy of GRACE was higher for the validation cohort than for the derivation cohort. One explanation might be that the follow-up periods of the 2 cohorts were different (the patients in the derivation cohort were followed for a mean duration of 32 months; the patients in the validation cohort received a 1-year follow up). Another study showed a similar finding, the longer the follow-up, the smaller the C-statistic [26].

The CADILLAC risk score was also found to be independently associated with the long-term mortality of AMI patients undergoing PCI (derivation cohort: HR = 1.334, 95% CI 1.243–1.432, *p* < 0.001; validation cohort: HR = 1.394, 95% CI 1.207–1.611, *p* < 0.001). Also, as a categorical variable, a simplified integer CADILLAC risk score could indicate risk stratification for patients with different scores (Table 2 and Figure 6). In our study, the CADILLAC risk score still showed good prognostic accuracy with good calibration for both the validation and derivation cohorts (C-statistics: 0.734, 0.731, respectively; H-L *p*-value = 0.946, 0.107, respectively) (Tables 3 and 5). In contrast with the GRACE risk scoring system, the CADILLAC risk scoring system was based on 2 multicenter randomized AMI trials of patients undergoing primary PCI, and contains both clinical and angiographic variables. Although it was not derived from ‘real-world’ data, the angiographic variables have been confirmed as important prognostic information [27, 28]. The CADILLAC risk score was also verified by other external cohorts [26, 27, 29, 30]. In our study, the CADILLAC risk score also showed good prognostic accuracy for both cohorts. Interestingly, unlike the GRACE risk score, the CADILLAC risk score yielded almost the same C-statistics for both cohorts. Another study by Brkovic et al found that the SYNTAX score, which grades the complexity of coronary angiographic anatomy, improved the prognostic performance of the GRACE risk score, but not the CADILLAC risk score. The long-term survival of patients after STEMI determined by the CADILLAC risk score mainly depended on clinical characteristics, myocardial function, and basic angiographic findings [27]. This might account for the consistency of the C-statistics for the CADILLAC risk scores of both of our cohorts. However, the CADILLAC risk scoring system performed worse in our study (C-statistics: 0.734 in the derivation cohort; 0.731 in the validation cohort) than in the derivation and validation cohorts from the CADILLAC risk scores [11], respectively, with C-statistics of 0.79 and 0.78, respectively. The most important reason for the discrepancy in results is the difference between the participants of the the CADILLAC risk scores and the participants of our study. The CADILLAC risk score was only derived from patients with STEMI undergoing primary PCI, and patients with NSTEMI, cardiogenic shock, and other severe diseases were excluded [11]. In contrast, our study was based on ‘real-world’ patients with AMI who underwent PCI.

### Limitations

This study had several limitations. Firstly, the cohorts were both from a single center. Our study only included AMI patients undergoing PCI, and did not include patients receiving conservative medical treatment only. The derivation cohort was retrospective. So, potential confounders and selection bias could not be completely adjusted. Secondly, compared with the original study cohorts [5–9], our study cohorts were relatively small. Thirdly, our study could not test other powerful mortality prediction models because of missing data. For example, our cohorts had no data for the Zwolle risk score on ischemia time in NSTEMI patients [31]; for the TIMI risk score, use of aspirin over the last 7 days in NSTEMI patients [32]; for the PURSUIT risk score, worst Canadian Cardiovascular Society (CCS) class of the 6 weeks [33]; for the PAMI risk score, left bundle branch block [34]. Therefore, comparing the ACTION risk score with other risk scores becomes difficult. Fourthly, our study found that the C-statistics of all 3 risk scores were lower than those in the original cohorts. This is mainly accounted for by the very low mortality in our study. The lower mortality in our cohorts is due to the lack of adequate medical emergency infrastructure and a large number of low-income people. In remote districts or rural areas, many high-risk AMI patients cannot be rapidly transferred to a hospital that offers PCI because of lack of adequate medical emergency infrastructure. In addition, high-risk AMI patients usually need more treatment time and more expensive treatment, and their outcomes are worse. Under the conditions described, low-income, high-risk AMI patients usually refuse expensive PCI, and request conservative medical treatment only. Finally, we did not test the predictive ability of the updated ACTION risk model for in-hospital mortality and excluded patients who died during the index hospitalization, since the updated ACTION risk model was originally designed for predicting in-hospital mortality.

## MATERIALS AND METHODS

### Study Design and Setting

### Derivation Cohort and Validation Cohort

We performed this study using 2 independent cohorts. The derivation cohort consisted of a retrospective cohort study for which the rationale and design were previously described [12]. In brief, from January 1, 2010 to October 31, 2014, consecutive AMI patients, who were hospitalized and underwent PCI at a large hospital in Northeast China (Shengjing Hospital of China Medical University, Shenyang, China), were included. The clinical and procedural data of all cases were obtained by the investigators from electronic medical records and a picture archiving and communication system (PACS), which stores data from interventional imaging and the surgical records of PCI cases. Left ventricular ejection fractions (LVEFs) were determined during hospitalization by echocardiography. Thrombolysis in myocardial infarction (TIMI) flow scores were determined as defined previously [13]. The initial cohort consisted of 2060 patients. The following patients were then excluded: (1) missing ACTION, GRACE, or CADILLAC risk scores (80 cases), (2) lost to follow up (62 cases), (3) died during the index hospitalization (17 cases). The final cohort consisted of 1901 AMI patients undergoing PCI. Clinical follow-up was performed in October 2015 by phone interviews with the patient's general practitioner/cardiologist, patient him-/herself, or patient's family. All patients were followed for a mean duration of 32 months (12 to 67 months).

The validation cohort consisted of an ongoing, prospective, observational cohort–P-PUSH (prospective evaluation of prognosis of Pci patients) using network data from Shengjing Hospital of China Medical University. The P-PUSH database contained comprehensive clinical and procedural data from all consecutive AMI patients who underwent PCI from January 1, 2015, at Shengjing Hospital of China Medical University. We constructed patient care report forms (CRFs) that contained 332 discrete items that were subdivided, and included the following: demographic characteristics, past history, clinical characteristics on admission, laboratory measurements, procedure-related complications, and relevant therapeutic agents. The attending physician used the electronic medical records to complete the CRFs after discharge. The diagnoses of patients were classified by attending cardiologists according to guidelines [1–4]. LVEFs were determined during hospitalization by echocardiography. TIMI flow scores were determined as defined previously [13]. Prospective clinical follow-up after discharge was performed regularly during hospital visits or phone interviews with the patient's general practitioner/cardiologist, patient her-/himself, or his or her family in all participants. All events were adjudicated and classified by 2 cardiologists. The exclusion criteria of the validation cohort included the following: (1) missing ACTION, GRACE, or CADILLAC risk scores (21 cases), (2) lost to follow up (60 cases), (3) died during the index hospitalization (5 cases). The final validation cohort consisted of 728 AMI patients who underwent PCI and were followed for 1 year, from January 1, 2015 to January 1, 2016.

All-cause mortality was identified from the patients’ medical records or each patient's referring hospital physician. All events were validated by 2 independent physicians. This study complied with the Declaration of Helsinki, and the Shengjing Hospital of China Medical University Research Ethics Committee approved the research protocol. Written informed consent was obtained from all participants.

### Participants and procedures

AMI was defined according to current guidelines [1–4]. Briefly, non-ST-segment elevation myocardial infarction (NSTEMI) was defined as follows: (1) chest discomfort or anginal equivalent, (2) ST-segment depression, transitory ST-segment elevation or prominent T-wave inversion, and (3) positive biomarkers of necrosis (creatine kinase-myocardial band, cardiac troponin-I); ST-segment elevation myocardial infarction (STEMI) was defined as (1) duration of chest pain less than 12 hours from onset of pain to time of catheterization, (2) notable ST-segment elevation (at least 0.1 mV in 2 or more standard leads or at least 0.2 mV in 2 or more contiguous precordial leads), or (3) a new left bundle branch block.

PCI was undertaken according to recommendations of current guidelines [1–4]. The use of aspiration thrombectomy and glycoprotein IIb/IIIa inhibitor was decided by the responsible cardiologist. Based on the current guidelines, the performing cardiologist administered periprocedural and postprocedural antiplatelet treatments and other cardiovascular agents [1–4]. The updated ACTION, GRACE, and CADILLAC risk scores were determined as described previously [7, 9–11]; and patients were stratified based on risk scores, as follows: updated ACTION risk score, very-low-risk group (risk score < 30), low-risk group (risk score 30 to 39), intermediate-risk group (risk score 40 to 49 ), high-risk group (risk score 50 to 59), very-high-risk group (risk score > 59); GRACE risk score, low-risk group (risk score ≤ 88), intermediate-risk group (risk score 89 to 118 ), and high-risk group (risk score > 118); CADILLAC risk score, low-risk group (risk score ≤ 2), intermediate-risk group (risk score 3 to 5), and high-risk group (risk score > 5).

### Statistical analysis

Quantitative variables were represented as means ± standard deviation (SD) or medians (interquartile range [IQR]), and categorical variables were represented as counts and proportions (%). Cox proportional hazards regression modeling by the forward stepwise procedure was used to analyze the effect of variables on event-free survival. Results were reported as hazard ratios (HRs) with associated 95% confidence intervals (CIs). The performance of the various risk scores was assessed by indices of calibration and discrimination. Each risk score was entered into a logistic regression model to obtain individual risk probability of all-cause death. The Hosmer-Lemeshow (HL) statistic from regression modeling was used as an indicator of goodness-of-fit of each risk score and to assess calibration ability [14]. In the HL Goodness-of-Fit test, higher *p* values indicate better calibration. The discriminative power of each risk score was assessed by the concordance (C) statistic, which was defined by the area under the receiver operating characteristic (AUROC) curve in relation to long-term all-cause mortality [15]. The C statistics for the 3 risk scores were compared by a nonparametric test developed by DeLong et al. [16, 17], with the use of MedCalc software for Windows, version 11.4.2.0 (MedCalc Software, Mariakerke, Belgium). All tests were two-sided, and statistical significance was defined as *p* < 0.05. Statistical analysis was performed by SPSS version 19 (SPSS Inc., Chicago, Illinois, USA).

## CONCLUSIONS

The updated ACTION risk model predicts the long-term mortality of patients with AMI who undergo PCI. Despite their development over a decade ago, the GRACE and CADILLAC risk models still maintain good performance for predicting the long-term risk of death in AMI patients undergoing PCI from 2 recent cohorts. The discriminatory performance of the updated ACTION risk model for predicting long-term mortality was similar to the discriminatory performances of the GRACE and CADILLAC scoring systems.
